# A human mitochondrial poly(A) polymerase mutation reveals the complexities of post-transcriptional mitochondrial gene expression

**DOI:** 10.1093/hmg/ddu352

**Published:** 2014-07-09

**Authors:** William C. Wilson, Hue-Tran Hornig-Do, Francesco Bruni, Jeong Ho Chang, Alexis A. Jourdain, Jean-Claude Martinou, Maria Falkenberg, Henrik Spåhr, Nils-Göran Larsson, Richard J. Lewis, Lorraine Hewitt, Arnaud Baslé, Harold E. Cross, Liang Tong, Robert R. Lebel, Andrew H. Crosby, Zofia M. A. Chrzanowska-Lightowlers, Robert N. Lightowlers

**Affiliations:** 1 Wellcome Trust Centre for Mitochondrial Research, Institute for Ageing and Health,; 2 Institute for Cell and Molecular Biosciences and,; 3 Wellcome Trust Centre for Mitochondrial Research, Institute for Cell and Molecular Biosciences, Newcastle University Medical School, Newcastle upon TyneNE2 4HH, UK,; 4 Department of Biological Sciences, Columbia University, New York, NY 10027, USA,; 5 Department of Cell Biology, University of Geneva, 30 Quai Ernest-Ansermet, 1211 Genève 4, Switzerland,; 6 Department of Biochemistry and Cell Biology, University of Göteborg, Box 440, 40530 Göteborg, Sweden,; 7 Max Planck Institute for Biology of Ageing, Gleueler Strasse 50a, D-50931 Cologne, Germany,; 8 Department of Ophthalmology, University of Arizona School of Medicine, Tucson, AZ85711, USA,; 9 Center for Behavior, Development, and Genetics, Medical Genetics, SUNY Upstate Medical University, Syracuse, NY13210, USA and; 10 Molecular Genetics, University of Exeter Medical School, Royal Devon and Exeter Hospital, Barrack Road, ExeterEX2 5DW, UK

## Abstract

The p.N478D missense mutation in human mitochondrial poly(A) polymerase (mtPAP) has previously been implicated in a form of spastic ataxia with optic atrophy. In this study, we have investigated fibroblast cell lines established from family members. The homozygous mutation resulted in the loss of polyadenylation of all mitochondrial transcripts assessed; however, oligoadenylation was retained. Interestingly, this had differential effects on transcript stability that were dependent on the particular species of transcript. These changes were accompanied by a severe loss of oxidative phosphorylation complexes I and IV, and perturbation of *de novo* mitochondrial protein synthesis. Decreases in transcript polyadenylation and in respiratory chain complexes were effectively rescued by overexpression of wild-type mtPAP. Both mutated and wild-type mtPAP localized to the mitochondrial RNA-processing granules thereby eliminating mislocalization as a cause of defective polyadenylation. *In vitro* polyadenylation assays revealed severely compromised activity by the mutated protein, which generated only short oligo(A) extensions on RNA substrates, irrespective of RNA secondary structure. The addition of LRPPRC/SLIRP, a mitochondrial RNA-binding complex, enhanced activity of the wild-type mtPAP resulting in increased overall tail length. The LRPPRC/SLIRP effect although present was less marked with mutated mtPAP, independent of RNA secondary structure. We conclude that (i) the polymerase activity of mtPAP can be modulated by the presence of LRPPRC/SLIRP, (ii) N478D mtPAP mutation decreases polymerase activity and (iii) the alteration in poly(A) length is sufficient to cause dysregulation of post-transcriptional expression and the pathogenic lack of respiratory chain complexes.

## INTRODUCTION

The human mitochondrial genome, mtDNA, encodes 13 polypeptides, all of which are essential for the coupling of cell respiration to ATP production (1). These proteins arise from the intra-mitochondrial translation of mt-mRNAs processed from large polycistronic precursors, which are mostly matured by the addition of short (∼50 nt) poly(A) tails (2,3). Adenylation is necessary to complete the termination codon of seven open-reading frames, but the rationale for why human mt-mRNAs are poly- rather than oligo-adenylated remains unclear. Although polyadenylation of transcripts has been almost universally maintained in cells and organelles, its function varies dramatically (4,5). For example, in the eukaryotic cytosol, the polyadenylation of mRNA permits association with poly(A)-binding protein (PABP) family members, leading to an increase in mRNA stability (6,7). Further, the presence of a poly(A) tail and PABPs allows interactions with factors that bind to the 5′ methylguanylate cap structure of mRNAs thereby stimulating translation initiation (8). Conversely, polyadenylation of RNA in various eubacteria can promote degradation and regulate quality control of more stable tRNA or rRNA (9). Furthermore, variations in polyadenylate function are also apparent between organelles. Yeast lack a mitochondrial poly(A) polymerase (mtPAP), and no mitochondrial mRNA carries a 3′ post-transcriptional extension (4,10), whereas polyadenylation of plant and algal organellar RNA follows the eubacterial pattern and stimulates decay (11,12). Trypanosome mitochondria are yet more complicated. Short (15–20 nt) oligo(A) tails are added to pre- or fully edited RNA and are necessary to promote stability, whereas longer U/A extensions of 200–300 nt designate the mRNA as ready for translation (13). It has long been known that human mt-mRNA can be polyadenylated (3), but even after numerous attempts to determine its exact role, the function of this modification is still unclear. A number of different approaches by various groups have been used to address this question. These include siRNA-mediated depletion of transcripts encoded by the *PAPD1* gene, now termed *MTPAP* in the new nomenclature (14–16), occlusion or degradation of poly(A) tails by targeting cytosolic PABP or poly(A) nuclease to the mitochondrion (17), and overexpression of the mitochondrial deadenylase PDE12 (18). In each case, mt-mRNAs were analysed and different effects on stability were observed, dependent on the transcript analysed.

All these methods, however, introduced some level of artificial genetic manipulation, each of which can be prone to artefact and consequently difficulties in interpretation of resulting data. In contrast, we were invited by an Old Order Amish family to investigate the cause of a profound form of spastic ataxia with optic atrophy in a subset of family members. Samples from these patients indicated that their clinical condition resulted from a homozygous p.N478D missense mutation in the *MTPAP* gene (19). Our initial studies of mRNA transcripts obtained from family blood samples were able to confirm that the mutation caused a loss of polyadenylation in the mt-mRNA species analysed. More recently, using fibroblast cell lines from affected as well as unaffected family members, we have been able to undertake more in-depth analysis into the consequences of non-polyadenylation of mt-mRNAs.

## RESULTS

### The p.N478D mtPAP mutation causes a defect in oxidative phosphorylation

**Figure 1.**
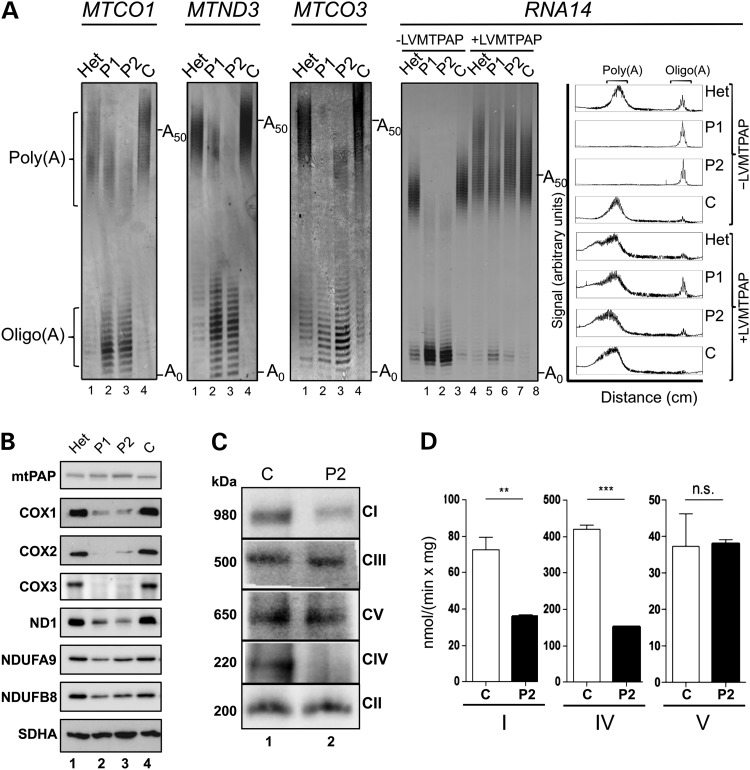
The MTPAP 1432A>G mutation causes defective mt-mRNA polyadenylation and a respiratory chain deficiency. (**A**) RNA was isolated from patient (P1 and P2) and control (Het, unaffected heterozygote; **C**, unrelated control) fibroblasts before (lanes 1–4) or after (lanes 5–8) transduction with a wild-type *MTPAP* transgene (±LVMTPAP). The length of the mRNA poly(A) tail was assessed by MPAT in the four transcripts indicated. Representative gels depict fluorescently-labelled products separated through a 10% denaturing polyacrylamide gel and visualized by laser scanning. Zero extension (A_0_) is the position of migration predicted post-3′ processing of the transcript prior to any addition. A_50_ indicates the position of a poly(A) of 50 nt. Densitometric profiles of the signal from the *RNA14* MPAT are presented in the far right panel. (**B**) Cell lysates (40 µg) isolated from patient and control fibroblasts were separated via 12% denaturing SDS–PAGE, and immunoblotting was performed. The images are representative of data using antibodies targeting mtPAP and OXPHOS subunits (listed in materials and methods). Detection was by ECL+ and Biorad ChemiDoc MP imaging system. (**C**) Mitochondria (40 µg) isolated from patient and control fibroblasts were analysed by Blue Native PAGE (4.5–16%). Each of the OXPHOS complexes was decorated using primary antibodies targeted to NDUFA9 (CI), Core 2 subunit (CIII), α-subunit (CV), the holoenzyme (CIV) and SDHA (CII). Sizes of the detected complexes are indicated to the left of panels, and the complex identities are shown on the right. (**D**) The activities of OXPHOS complexes I, IV and V were determined in mitochondria isolated from patient (black) and control (white) fibroblasts. Activities are expressed as nmol rotenone-sensitive NADH oxidized/min/mg mt-protein (CI), nmol reduced cytochrome *c* oxidized/min/mg mt-protein (CIV) and NADH oxidized/min/mg mt-protein (CV). *N* = 4, errors bars indicate ±SD. Student *t*-test (ns *P < 0.05; **P < 0.01; ***P < 0.001).

Our initial investigations were limited to the analysis of blood samples from members of a large Old Order Amish family with multiple children suffering from a progressive and autosomal recessive neurodegenerative disorder. Genomic analysis revealed a candidate pathogenic mutation in *MTPAP*, the gene encoding mtPAP. We therefore performed mitochondrial poly(A) tail assays of representative mitochondrially encoded transcripts from whole blood. These confirmed an mt-mRNA polyadenylation defect associated with the c.1432A>G (p.N478D) mutation in clinically affected members, but no further studies were possible at that time (19). Here, we present investigations on skin fibroblast cell lines from three family members, two affected individuals homozygous for the mutation (P1 and P2) and one unaffected heterozygote sibling (Het). Initially, it was important to confirm that the polyadenylation defect was recapitulated in the cultured fibroblasts. RNA was isolated, and four mt-mRNAs were subjected to the mitochondrial poly(A) tail length assays as described previously (20). For each mt-mRNA analysed, a lack of polyadenylation was apparent in the pathogenic homozygote lines (P1 and P2), concomitant with an increase in oligoadenylated species (Fig. 1A lanes 2 and 3). The heterozygote behaved similarly to the control line, although there was evidence of a mild increase in oligoadenylated mRNA (Fig. 1A lane 1 cf lane 4). Homopolymeric oligoadenylation was confirmed by sequence analysis of MPAT-derived species for *MTCO1* (data not shown). We then assessed whether the marked loss of polyadenylation caused any phenotypic consequences. Cell growth rate was examined first, using either glucose- or galactose-based media. Galactose is a carbon source that forces cells to use oxidative phosphorylation (OXPHOS). The growth defect seen in the homozygote mutant lines was more pronounced on this substrate than on glucose (Supplementary Material, Fig. S1). Cell lysates from the two patient cell lines, the unaffected sibling (Het) and one unrelated control (C) were then prepared and subjected to western blot (Fig. 1B). No observable difference in the steady-state levels of mtPAP could be detected, showing the p.N478D mutation did not affect the stability of the protein. In contrast, in the homozygote p.N478D lines, a substantial decrease was apparent in the mitochondrially encoded proteins of the respiratory chain, ND1, a component of complex I and COX1–3, members of complex IV. NDUFB8, although nuclear encoded, was also present at decreased steady-state levels as it is sensitive to complex I assembly. As expected, no decrease in complex II (SDHA) was observed consistent with all its components being nuclear encoded. Analysis of the fully assembled OXPHOS complexes by blue native gels (Fig. 1C) reflected the severe decrease seen in components of complexes I and IV, and this was further mirrored in the enzymatic activities (Fig. 1D). Intriguingly, complexes III and V appeared unaffected. Taken together, these data are consistent with a selective defect of mitochondrial gene expression.

To confirm that the mtPAP p.N478D mutation was responsible both for the loss of mt-mRNA polyadenylation and the consequent reduction in complexes I and IV, we designed a lentiviral vector to express wild-type mtPAP constitutively. Following transduction and selection of the four cell lines (P1, P2, Het and C), lysates and RNA were prepared. Expression of wild-type mtPAP (+LVMTPAP) in the homozygote mutant cell lines was sufficient to restore steady-state levels of mtDNA gene products at both the mRNA and protein level (Supplementary Material, Fig. S2A and B). Further, analysis of various mt-mRNAs showed a restoration of poly(A) tail for lines P1 and P2 (*RNA14*, Fig. 1 lanes 2, 3 cf 6, 7; *MTND3*, Supplementary Material, Fig. S2C lanes 2, 3 cf 6, 7). In all four cell lines transduced with wild-type mtPAP, the final tail length exhibited a slightly increased median length compared with the untransduced controls (lanes 1, 4 cf 5–8 for both *RNA14* in Fig. 1A and *MTND3* in Supplementary Material, Fig. S2C).

### Aberrant mtPAP induces significant effects on the steady-state levels and translation of mitochondrial mRNA species

**Figure 2.**
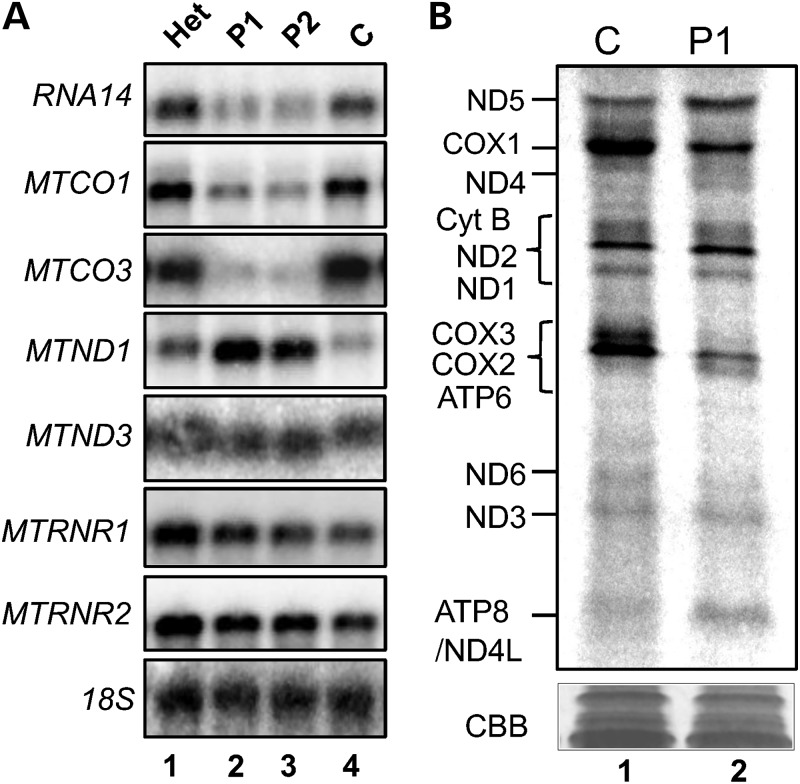
The p.N478D mutation in mtPAP affects steady-state levels and translatability of mtDNA-encoded transcripts. (**A**) RNA was isolated from control and patient fibroblasts, and steady-state levels of mitochondrial rRNA and mRNA were assessed by northern blotting (4 µg), using the probes indicated. A probe against the *18S* rRNA transcript was used as a loading control. The blot is representative of data from three independent RNA isolations. (**B**) *De novo* protein synthesis of mitochondrially encoded proteins was analysed in control and patient fibroblasts. Cells were incubated with [^35^S]-methionine/cysteine for 1 h in the presence of the cytosolic translation inhibitor emetine dihydrochloride (100 µg/ml). Equal amounts of whole cell lysate (50 µg) were separated via 15–20% denaturing SDS–PAGE and translation products visualized by autoradiography. Individual polypeptides were designated by their mobility (21). The protein profiles are representative of data derived from three independent experiments.

Depletion of mtPAP, mediated by siRNAs targeting the *MTPAP* transcript, has been shown by several groups to result in transcript-specific effects on the steady-state levels of mt-mRNAs in cultured human cell lines (14–16). In the homozygote patient lines (Fig. 2A lanes 2 and 3), we also saw transcript-specific effects, where steady-state levels of *RNA14* and all *MTCO* were decreased, *MTND1* increased and *MTND3* unaffected. A similar trend had been noted when mt-transcripts lacked polyadenylated termini as a consequence of the poly(A) tails being removed by a cytosolic poly(A)-specific ribonuclease that had been targeted to mitochondria (17). To determine what effect these altered steady-state levels, caused by the mutant mtPAP, had on the translation of these transcripts, *de novo* metabolic labelling was performed. Autoradiographic data analysis of the homozygote compared with the control indicated that there were varied but reproducible differences in the amount of translation products from different transcripts (Fig. 2B). There was an evident decrease in *de novo* synthesis of the components of the COX2/3 ATP6 triplet, which was in contrast to the increase in ND5 and ND2. Those transcripts that showed decreased stability, such as *MTCO3*, showed a concomitant decrease in translation product. However, there was no consistent correlation between transcript level and translation as the dramatic increase in stability of *MTND1* (Fig. 2A) did not result in a concomitant increase in ND1 (Fig. 2B).

### Mutant and wild-type mtPAP are found in mitochondrial RNA granules

**Figure 3.**
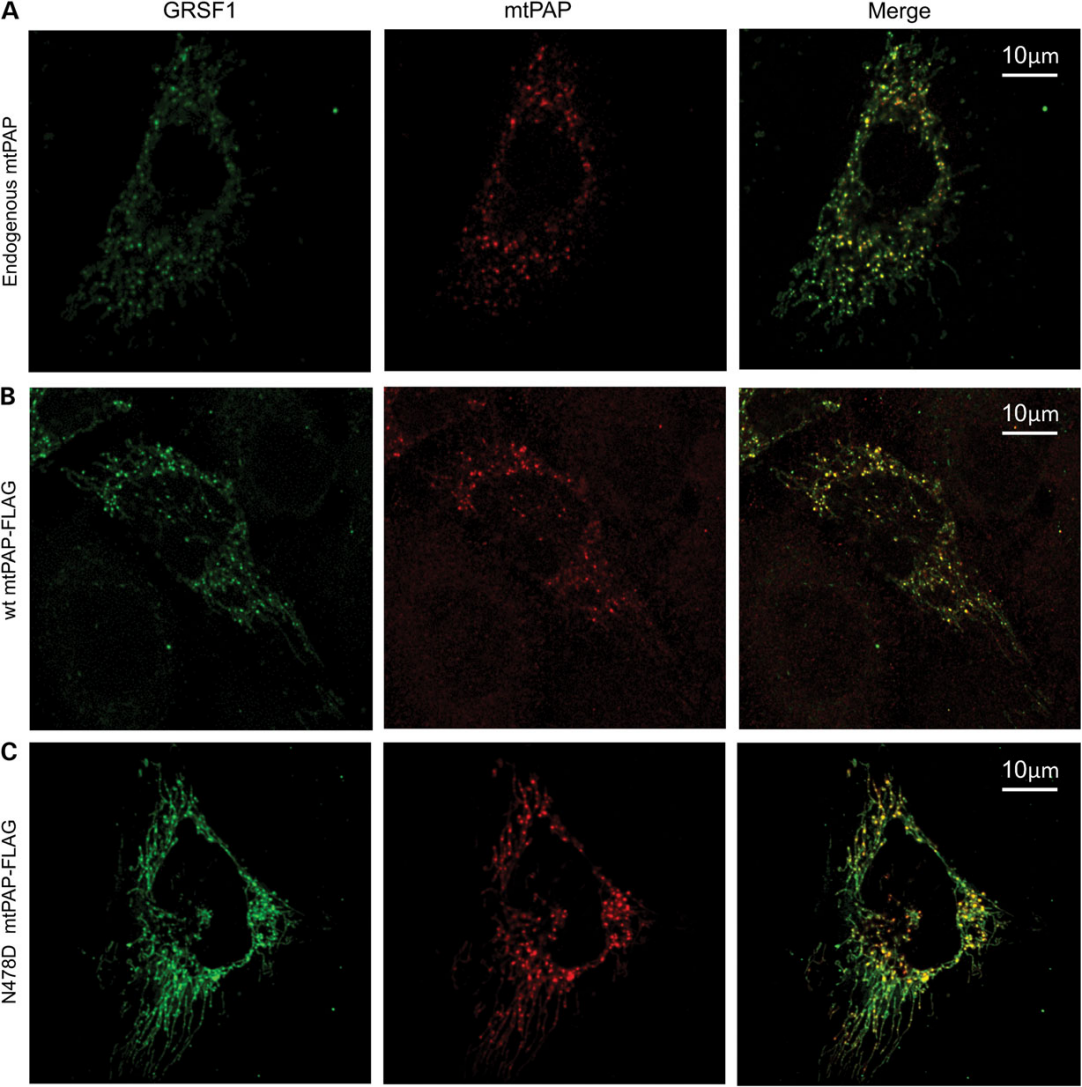
Both wild-type and mutant mtPAP co-localize with mitochondrial RNA granules. (**A**) HeLa cells expressing an HA-tagged version of GRSF1 isoform 1 (GRSF1) were immunolabelled with anti-HA and anti-mtPAP (endogenous) antibodies and then analysed by confocal microscopy. Images were merged to determine co-localization. (**B**) HeLa cells transfected to express both HA-tagged GRSF1-HA and a C-terminal FLAG-tagged wild-type mtPAP were immunolabelled using anti-HA and anti-FLAG antibodies and analysed by confocal microscopy. Images were merged to determine co-localization. (**C**) Similar confocal analysis was performed on HeLa cells transfected to express GRSF1-HA and the p.N478D mutant version of mtPAP-FLAG. Immunolabelling and image analysis was as described in (B).

It has been shown previously that mitochondrial RNA is processed in distinct foci referred to as mitochondrial RNA granules (MRGs) (22). We posited that the maturation of mitochondrial mRNA may also occur in MRGs. Consequently, we performed immunocytochemistry to detect mtPAP (endogenous, Fig. 3A) and a marker for the MRGs, namely GRSF-1. As shown in Figure 3A, both antibodies revealed discrete co-localizing foci within the mitochondrial network, demonstrating that mtPAP is indeed present in MRGs. Repeat experiments with FLAG-tagged wild-type mtPAP (Fig. 3B) also showed this punctate distribution. We then wished to determine whether the p.N478D mutation led to any loss of mtPAP localization. As seen in Figure 3C, the p.N478D mtPAP also showed a similar pattern of co-localization, consistent with the mutant being present in MRGs.

### Activity of the p.N478D-mutant mtPAP is severely compromised

**Figure 4.**
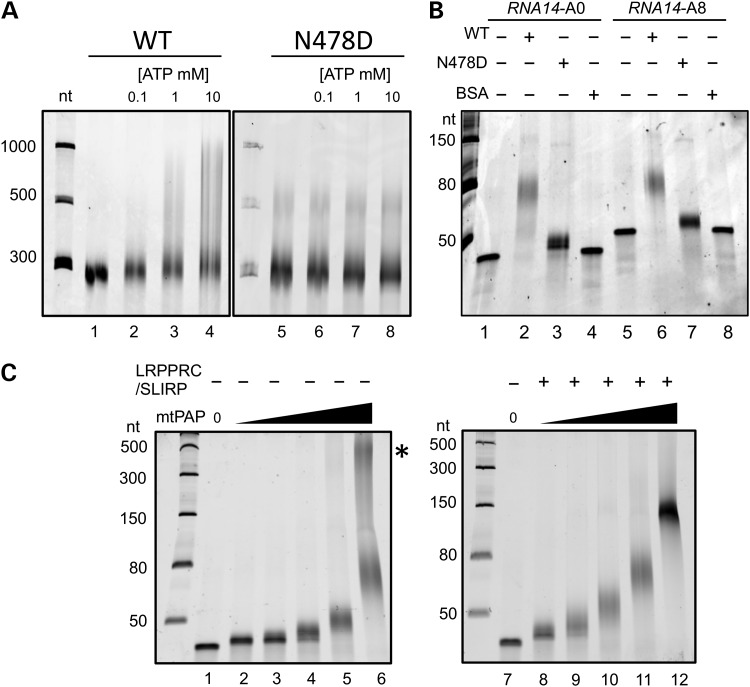
*In vitro* polyadenylation activity of mtPAP. (**A**) Polyadenylation activity of recombinant wild-type (WT; lanes 2–4) and mutant (p.N478D; lanes 6–8) mitochondrial poly(A) polymerase (0.55 µm) was determined with increasing ATP concentrations. The RNA substrate was an unadenylated 277-nt 3′ fragment of *MTND3* (0.25 µm). The right hand panel contains an IVT RNA artefact (500 nt) present in the absence of mtPAP (lane 5). Reactions were quenched with 90% formamide/1× TBE, separated through a 6% polyacrylamide/8.3 m urea gel, then stained with SYBR gold and visualized by scanning with a Typhoon FLA 9500 instrument. (**B**) Short RNAs (0.25 µm) corresponding to the final 40 nucleotides of *RNA14* with (A8, lanes 5–8) or without (A0, lanes 1–4) an oligo(A8) addition were used as templates for polyadenylation by recombinant wild-type (WT; lanes 2 and 6) or mutant (p.N478D; lanes 3 and 7) mtPAP (0.55 µm). An equal amount of BSA was added in a parallel experiment as a control (lanes 4 and 8). Products were separated through 15% polyacrylamide/8.3 m urea gel and visualized as in (A). (**C**) Increasing amounts of wild-type mtPAP (34 nm to 0.55 µm) were added to the short *RNA14*A0 (0.25 µm) template in the presence (lanes 8–12) or absence (lanes 2–6) of LRPRRC/SLIRP complex. A higher molecular species (*) of varying intensity was observed with wild-type mtPAP. Products were separated and visualized as in (B).

The p.N478D mtPAP mutation did not affect either the stability or the location of the enzyme necessitating further investigations to characterize the effect of this mutation on mtPAP. The crystal structure of this non-canonical poly(A) polymerase has been solved (23) revealing that the protein dimerises but lacks a classic RNA-binding domain. The region encompassing p.N478, however, was disordered in the crystal. To determine whether the p.N478 mutation influences the dimer formation, we overexpressed and purified both the wild-type and mutant enzyme for use in *in vitro* studies. Both wild-type and mutant mtPAP were isolated to electrophoretic purity and subjected to gel filtration analysis. As shown in Supplementary Material, Fig. S3, both mutant and wild-type mtPAP form dimers indicating that the p.N478D mutation does not affect the overall fold or the oligomeric state of the protein. The mutation, therefore, must affect mtPAP function directly, because the N478 region does not contribute to the dimer interface (23). We subsequently compared polyadenylation activities of the two enzymes *in vitro*. Initially, we generated long RNA substrates [terminal 277 nt of *MTND3* or 248 nt of *RNA14*, with or without oligo(A8)]. The wild-type enzyme extended from both templates irrespective of the oligoadenylation status. The mutant protein, however, was unable to discernibly extend unadenylated *MTND3* substrate (Fig. 4A). Using such a large substrate under these electrophoresis conditions, however, meant that it was not possible to determine whether p.N478D was entirely inactive or only able to oligoadenylate, as could be seen for mt-mRNA in the mutant cell lines (Fig. 1A). The absence of any activity would suggest that another poly(A) polymerase would have to be present to add the oligo(A) modification. We therefore designed a number of short (40 nt) RNA substrates to mimic either internal sections of *MTCO1* and *MTND3* or the native 3′ end of *RNA14* post-processing. The latter substrate was synthesized with or without an oligo(A8) 3′ terminal extension to establish whether mtPAP had a different preference for (i) correctly processed 3′ termini compared with any free 3′ end and (ii) to see whether mtPAP only used pre-oligoadenylated transcripts as a template. Saturating levels of wild-type mtPAP were able to extend ∼40 nt from the 3′ terminus of both the internal *MTND3* sequences (Supplementary Material, Fig. S4) and also correctly processed *RNA14* with or without oligo(A8) ends (Fig. 4B lanes 2 and 6). Thus, the wild-type mtPAP demonstrated no preference for 3′ terminal sequence of the RNA substrate. In addition to the main population of extended products, there was a second, much longer population. The ratio of this product (Fig. 4C lane 6, *) to the main product varied between experiments. In contrast, the mutant mtPAP showed severely compromised activity, where the extension achieved on either template, with or without oligo(A8), was only 5–10 nt (*RNA14* shown in Fig. 4B lanes 3 and 7). The mutant protein was not able to generate longer extensions even in prolonged -h incubations. To determine whether the p.N478D mutation affected polymerase processivity, it was first necessary to identify whether mtPAP was distributive or processive under these *in vitro* conditions. The extension assay was therefore performed under conditions where the enzyme/RNA substrate ratio fell below 1 : 1. Figure 4C shows that the wild-type enzyme does not fully extend the subset of RNA substrate to which it has bound, indicating that under these *in vitro* conditions, it is distributive, thus precluding any effect of the mutation on enzyme processivity.

### LRPPRC and LRPPRC/SLIRP enhance poly(A) tail length extension

**Figure 5.**
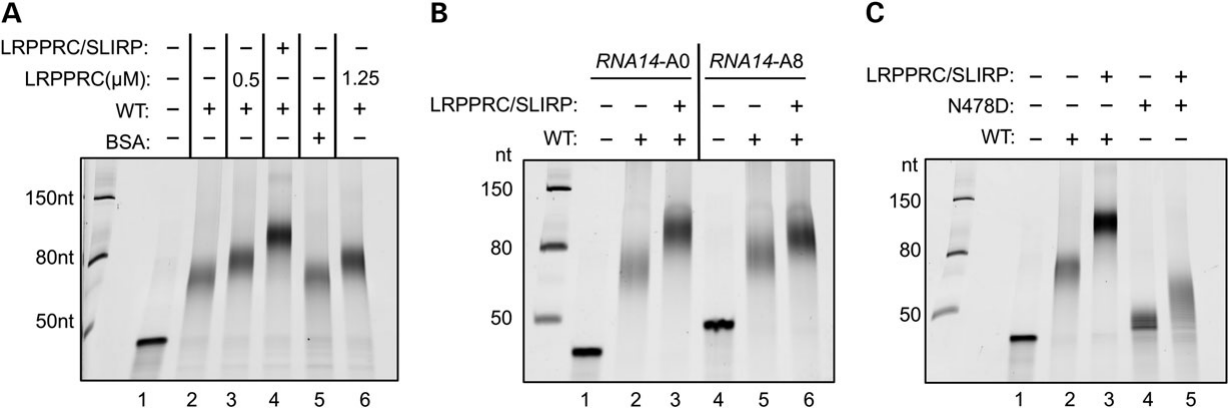
The LRPPRC/SLIRP complex modulates the polyadenylation activity of both wild-type and mutant mtPAP. (**A**) Polyadenylation activity was analysed using wild-type mtPAP (0.55 µm) incubated for 1h with the short *RNA14*A0 substrate (0.25 µm), alone (lane 2), with LRPPRC (0.5 µm lane 3; 1.25 µm lane 6), LRPPRC/SLIRP complex (0.5 µm, lane 4) or BSA (0.5 µm, lane 5). Products were separated and visualized as described earlier. (**B**) The effect of LRPPRC/SLIRP complex (0.48 µm) on polyadenylation by wild-type mtPAP (0.55 µm) of an unadenylated (A0, lanes 2 and 3) compared with an oligoadenylated (A8, lanes 5 and 6) *RNA14* substrate was analysed. Products were separated and visualized as described earlier. (**C**) The effect of LRPPRC/SLIRP complex (0.48 µm; lanes 3 and 5) on polyadenylation by p.N478D mutant (0.55 µm; lanes 4–5) poly(A) polymerase was compared with wild-type (0.55 µM; lanes 2–3) enzyme. The short unadenylated *RNA14*A0 substrate (0.25 µm) was used. Products were separated and visualized as described earlier.

Leucine-rich pentatricopeptide repeat-containing protein (LRPPRC) binds mature or unprocessed mitochondrial mRNA *in vivo* (24,25) and is often found associated with another much smaller RNA-binding protein, SLIRP (SRA stem loop interacting RNA-binding protein) (26). When the gene encoding LRPPRC or its homologue was deleted in mice or *Drosophila,* the resulting mt-mRNAs were found at low steady-state level, with reduced poly(A) tail lengths, and in many cases were aberrantly translated (27,28). Further, a recent study concluded that LRPPRC promoted mtPAP-mediated polyadenylation, possibly by resolving secondary structures at the 3′ termini of RNA substrates, facilitating access of mtPAP to unstructured 3′ ends (25). We therefore examined whether the presence of LRPPRC or the LRPPRC/SLIRP complex affected poly(A) extension by wild-type or mutant mtPAP. In the presence of wild-type mtPAP and LRPPRC (0.5 or 1.25 µm) poly(A) tail length was increased by a mean value of ∼12 nt (Fig. 5A cf lanes 2 and 3, 5 and 6). However, the extension was more pronounced on addition of the LRPPRC/SLIRP complex (Fig. 5A lane 4 cf lane 2), whereas SLIRP alone had only a minimal effect on mtPAP activity (data not shown). Assays were performed for 1 h, but increasing extension time to 2 h had only a minimal effect on maximal tail length (Supplementary Material, Fig. S4). Interestingly, a similar modulation of extension by LRPPRC/SLIRP was noted on an RNA substrate carrying eight adenylates (A8) residues at the 3′ terminus (Fig. 5B) precluding the increase in extension being due to LRPPRC/SLIRP resolving 3′ terminal structure. Assays performed with the p.N478D-mutant mtPAP together with the LRPPRC/SLIRP complex also resulted in a further, minor, extension of only 15–20 nt (Fig. 5C lanes 4 and 5), in comparison with an additional extension of ∼50 nt with wild-type mtPAP in the presence of complex (Fig. 5C cf lanes 2 and 3). Intriguingly, the longer population illustrated in Figure 4C lane 6 was in all cases resolved upon the addition of LRPPRC/SLIRP (Fig. 4C lane 12).

## DISCUSSION

The p.N478D mutation in mtPAP causes a severe progressive neurodegenerative disorder in all homozygous patients identified. The resultant cellular defect was identical in both of the patient cell lines analysed and manifested as a profound loss of mitochondrial OXPHOS complexes I and IV. Why such selectivity ? The mtPAP mutation caused selective variability in the stability of mt-mRNAs presumably due to their lack of polyadenylation. There have been several previous studies into the effects of deadenylation on mt-mRNA stability in human cultured cell lines, by using siRNA depletion of mtPAP in human cell lines (14–16), overexpression of a mitochondrial deadenylase PDE12 (18), or by the removal of poly(A) tails by targeting the poly(A)-specific ribonuclease PARN to mitochondria (17). A consensus pattern of stability now appears to be emerging. The lack of a poly(A) tail appears to result in significant and transcript-specific effects on mt-mRNA stability—*MTND1*, *MTND2* increased; *MTCO transcripts* decreased; *MTND3*, *MTND5*, *MTCYB* mildly or unaffected. A similar trend was noted for the patient homozygote cell lines, albeit that *RNA14* was less stable than controls. These effects on transcript stability are clear, and yet, there is no credible hypothesis to explain the transcript-specific variability. What is the effect of this variability on translation? Several recent publications have suggested a link between the polyadenylation status of mt-mRNA and translation (8,17,18). It has been known for many years that translation in the eukaryotic cytosol can be stimulated by the presence of a poly(A) tail by a mechanism promoted through interaction between protein complexes at the 5′ methylguanylate cap and the 3′ tail of the transcript (8). However, mitochondrial mRNA species do not possess a 5′ cap structure or any known 5′ associating complexes (29,30). Artificial deadenylation studies reported dramatic effects on mitochondrial protein synthesis, but these data were complicated by the forced deadenylation causing either the loss of termination codons in open-reading frames (17) or loss of the small mitoribosomal subunit itself (18). Both of these effects would be expected to decrease *de novo* protein synthesis.

None of the earlier studies assessed the effects of siRNA-induced mtPAP depletion on mitochondrial protein synthesis, although Nagaike *et al*. (15) did report marked effects on respiration, consistent with a lack of OXPHOS complexes. Our current studies show that *de novo* mitochondrial protein synthesis was perturbed in the p.N478D mtPAP homozygote cell lines, with certain species being increased (ND5), some decreased (COX1–3) and many unaffected (e.g. ND1–3), and OXPHOS was severely compromised. There was a degree of correlation between effects on mt-mRNA stability and translation, as seen for complex IV subunits, but others did not correlate well (e.g. ND1 and ND5). In other relevant studies, knockout of LRPPRC in mouse cardiac tissue and skeletal muscle resulted in the decreased stability of all mitochondrial transcripts except for the sole L-strand encoded product, *MTND6* (28). The remaining pool of transcripts retained only oligo(A) tails, and translation was markedly affected. Although *de novo* synthesis of most species was decreased as predicted from the effect on transcript stability, one translation product was in substantial excess. Following deletion in the fly of BSF, the homologue of LRPPRC, the initial pattern of severely compromised polyadenylation and steady-state levels of mt-mRNA was apparent and similar to the mouse model (27). However, the fly deletion differed as the transcript showed an additional problem of aberrant processing, and a marked increase in translation was noted, along with the production of novel translation products. This would argue that at least in flies, polyadenylation of mt-mRNA is not a prerequisite for translation. Finally, in human cultured cell lines under conditions where poly(A) tails were masked by mislocalization to the mitochondrion of the major cellular poly(A)-binding protein, PABPC1, a decrease in translation of all mt-mRNAs was seen (17). So, how does the loss of the poly(A) tail on human mt-mRNA result in such profound defects in assembly and stability of OXPHOS complexes? Taken together with several of the studies referenced earlier, it is clear that even relatively mild perturbations in *de novo* synthesis of mitochondrially encoded proteins can have major consequences for the assembly of OXPHOS complexes, possibly reflecting the intricate process of integrating the various subunits that are synthesized in different subcellular locations. However, this still does not explain the molecular basis of why the absence of the 3′ maturation event of polyadenylation could cause a modulation in protein synthesis that initiates at the other end of the molecule. Many groups contributed to resolving how polyadenylation functioned in the cytosol of eukaryotes to stimulate translation. Research in human mitochondria has the disadvantages of not being able to transfect the organelle with reporter constructs or to have a faithful *in vitro* system for analysing protein synthesis (31,32). It has therefore taken numerous groups, many years and many experimental approaches to try and understand the exact role of polyadenylation in the mammalian mitochondrion, and despite these efforts, we are not yet in a position to fully explain this observation.

What is the consequence of the p.N478D mutation in human mtPAP? We have shown that the mutated enzyme was correctly dimerized and folded and could function in extending 3′ termini, although the extension lengths were severely curtailed, indicating that the mutation caused a defect in but not a total loss of polyadenylation activity. This may explain the retention of short oligo(A) tails on mt-mRNAs in the homozygous patient cell lines. The mutation is located in the highly conserved fingers domain of mtPAP (23). In the absence of any classical RNA-binding domain in this non-canonical polymerase, it has been suggested that the interface of this fingers domain with the palm region may function in substrate binding, although the exact RNA-binding domain is unclear (5). Alternatively, another enzyme/complex may act in tandem with mtPAP to facilitate either 3′ end binding or processivity as both wild-type and mutant mtPAP act distributively in our *in vitro* assays, irrespective of LRPPRC/SLIRP addition. The hypothesis that there are more, as yet unidentified, binding partners is particularly appealing, given the poor activity of even the wild-type mtPAP when assayed, *in vitro*. We have been exploring the possibility that a co-factor is necessary for optimal mt-PAP activity, but our preliminary co-immunoprecipitation assays were unable to identify any candidates to date. Recently, however, a similar co-IP approach was used to identify two associating components, the RNA helicase Suv3p and the RNA-degrading enzyme, PNPase (33). The authors of this intriguing paper supply further evidence to suggest that a transient complex of these three proteins function *in vivo* to regulate the poly(A) tail lengths of human mt-mRNAs dependent on the ratio of inorganic phosphate to ATP in the mitochondrion.

Finally, how does the interplay between mtPAP and LRPPRC/SLIRP impinge on polyadenylation? It has previously been reported that on its own *in vitro*, mtPAP was unable to extend 3′ termini beyond short oligo(A) additions and required the presence of LRPPRC to promote extension (25). The authors of that study performed several experiments to show that LRPPRC promoted polyadenylation *in vitro*, suggesting that the complex acted by resolving double-stranded regions to reveal single-stranded RNA 3′ termini. We found some similarities to this impressive work, but our data differed in several respects. First, we found that mtPAP alone was able to extend from 3′ termini to produce poly(A) tails. Second, although we were able to show a consistent increase on the length of 3′ extension by the addition of the LRPPRC, the extension was markedly enhanced when LRPPRC was complexed with SLIRP. Third, we observed a consistent LRPPRC/SLIRP-mediated increase in poly(A) tail length even in the absence of 3′ secondary structure, arguing that the role of the complex cannot only be to resolve secondary structure. Absolute extension varied mildly with the different buffer conditions tested, but in all cases, the trends remained identical. This promotion of extension is intriguing. In addition to the extension of the major poly(A) species, a longer population of varying proportion (Fig. 4C lane 6) was lost on addition of the LRPPRC alone or complexed with SLIRP (Fig. 4C lane 12). The presence of LRPPRC/SLIRP therefore appeared to act as a molecular ruler. This interplay is reminiscent of a tripartite relationship that regulates poly(A) extension in the eukaryotic nucleus. The cleavage and polyadenylation-specific factor CPSF binds to RNA elements close to mRNA 3′ termini and stimulates poly(A) polymerase-mediated polyadenylation by interacting with the nuclear poly(A)-binding protein PABPN1 that coats the nascent poly(A) tail (34). When the tail reaches ∼250 nt, PABPN1 then acts to disrupt the interaction with CPSF, curtailing further extension. Although this similarity is interesting, it cannot be an exact model for mtPAP. PABPN1 breaks the processivity exhibited by nuclear PAP, and we have been unable to demonstrate processivity of mtPAP *in vitro*.

In summary, we have shown that the p.N478D mutation in mtPAP is pathogenic. The defect prevents the enzyme from efficiently polyadenylating mt-mRNA. The resultant loss of polyadenylation causes a differential modulation of steady-state levels of specific mt-mRNAs and perturbs mitochondrial protein synthesis leading to profound depletion of complexes I and IV of the respiratory chain, resulting in a form of spastic ataxia and optic atrophy.

## MATERIALS AND METHODS

### Cell culture and viral vectors

Primary human dermal fibroblasts were established from individuals either heterozygous or homozygous for the 1432A>G mutation in the *MTPAP* gene. Cell lines were immortalized by retroviral expression of the HPV-16 E6E7 gene. Cells were cultured in reagents purchased from Gibco Life Technologies; DMEM supplemented with 10% heat-inactivated foetal bovine serum, 50 U/ml penicillin, 50 µg/ml streptomycin and 50 µg/ml uridine (5% CO_2_ at 37°C). Wild-type expression of *MTPAP* was achieved by transduction with lentiviral particles (Genecopoeia) conferring puromycin resistance.

### Mitochondrial poly(A) tail assay

The (20) technique was modified to use AlexaFluor^®^ 647-labelled gene-specific nested forward primers instead of radiolabelled primers in the second round of PCR. The resulting PCR products were separated on a 10% polyacrylamide/8.3 m urea gel and directly imaged using a Typhoon FLA 9500 instrument (GE/Fujifilm).

### Metabolic labelling of mitochondrial translation

Essentially as described in (35) with a modification of a 1-h incubation with radiolabel, protein aliquots (50 µg) were separated by 15–20% (w/v) gradient SDS–PAGE. Radiochemical signal was detected (Typhoon FLA 9500 instrument GE/Fujifilm) and proteins identified by comparing their migration patterns against established data (21).

### RNA extraction and northern blotting

RNA was prepared by TRIZOL (Invitrogen) extraction following manufacturer's recommendations. Northerns were performed as described (36) and signals quantified by scanning with Typhoon FLA 9500 instrument (GE/Fujifilm).

### MTPAP cloning, and purification of wild type and p.N478D mtPAP

Human *MTPAP* (corresponding to residues 44–538) was sub-cloned into the pET28a vector (Novagen) generating an *N*-terminal 6× His fusion tag. The p.N478D mutant was created with the QuikChange kit (Stratagene). Overexpression of wild-type and p.N478D mtPAP in *Escherichia coli* BL21 Rosetta (DE3) cells (Novagen) was induced by overnight incubation with 0.5 mm IPTG (Melford) at 20°C. The soluble protein was eluted from nickel affinity gel (Sigma) with 50 mm Tris, pH 8.5, 300 mm NaCl and 250 mm imidazole, further purified by size exclusion chromatography and then concentrated in a buffer containing 50 mm Tris, pH 8.5, 300 mm NaCl and 5% (v/v) glycerol.

### Cloning and purification of LRPPRC and LRPPRC/SLIRP

Codon-optimized (DNA 2.0) DNA constructs corresponding to the mature form of human LRPPRC and amino acid 18–109 of SLIRP were cloned in a pCDFDuet-1 vector (Novagen) for co-expression. In addition, LRPPRC was cloned in pJexpress 401 (DNA 2.0). In both constructs, LRPPRC contains a TEV-cleavable 6× His fusion tag at the *N*-terminus. LRPPRC and LRPPRC/SLIRP were expressed and purified as previously described for the MTERF4-NSUN4 complex (37).

### Low resolution polyadenylation assay

RNA substrates were *in vitro* transcribed using the MAXIscript^®^ SP6 Kit (Ambion) to generate 248- and 277-nt transcripts mapping to the 3′ ends of *MTATP6* and *MTND3*, respectively. Polyadenylation reactions, product separation and visualization were performed as described for high-resolution assays.

### High-resolution polyadenylation assay

Single-stranded RNA mapping to the 3′ 40 nt of *RNA14*, with and without oligo(A_8_) tails, were purchased from Dharmacon. Polyadenylation reactions were carried out in 15 µl volume composed of 50-ng RNA substrate, 50 mm Tris, pH 8.0, 40 mm KCl, 1 mm DTT, 0.1 mm ATP, 10 mm MgCl_2_, 0.1 mm MnCl_2_ and 0.5 U/μl SUPERaseIn RNase inhibitor (Invitrogen). Wild-type mtPAP, p.N478D mtPAP, LRPPRC/SLIRP complex or LRPPRC was added at 0.5 µm each and incubated at 37°C for 1 h unless stated. RNA products were fractionated on 15% polyacrylamide/8.3 m urea gels, stained with SYBR Gold (Invitrogen) and imaged with a Typhoon FLA 9500 instrument (GE/Fujifilm).

### Analysis of proteins by immunoblot and BN-PAGE

Proteins were separated by standard SDS–PAGE and then immobilized on PVDF membrane (Immobilon-P, Millipore) by wet transfer (100 V, 1 h at 4°C) in Towbin buffer. Membranes were blotted with primary antibodies followed by HRP-conjugated secondary antibodies (Dako) and visualized by ECL Prime (GE Healthcare). One-dimension blue native gel electrophoresis (4.5–16% w:v) was performed as described in (38) with 40 µg of protein loaded per lane. Primary antibodies were from MitoSciences COX1 (MS404), COX2 (MS405), COX3 (MS406), SDHA (MS204), NDUFA9 (MS111), NDUFB8 (MS105), Complex V α-subunit (MS502), Complex III core 2 (MS304); Santa Cruz ATP8 (sc-84231), TOM20 (sc-17764), LRPPRC (sc-66844); mtPAP (GeneTex GTX70156); COX holoenzyme (gift from L. Nijtmans), ND1 (gift from A. Lombes).

### OXPHOS activity assays

The activities of individual respiratory chain complexes were determined in isolated mitochondria as previously described for complex I and IV (39) and for complex V (40).

### Immunofluorescence microscopy

HeLa cells were plated on coverslips and transiently transfected with a HA-tagged version of GRSF1 isoform 1 (GRSF1-HA), and FLAG-tagged wild-type or p.N478D mtPAP. Immunolabelling with polyclonal anti-HA, monoclonal anti-mtPAP or monoclonal anti-FLAG was as described (22). Imaging was performed using a Zeiss LSM700 confocal microscope.

## SUPPLEMENTARY MATERIAL

Supplementary Material is available at *HMG* online.

## FUNDING

This work was supported by The Wellcome Trust (096919/Z/11/Z) to R.N.L. and Z.C.L.; Pathological Society of Great Britain and Ireland (PhD 2010/04) to Z.C.L. as a PhD Scholarship funding for W.C.W.; Deutsche Forschungsgemeinschaft postdoctoral Fellowship (GZ: HO 3326/2-1 AOBJ:584841) to H.T.H.D. and US NIH grant (R01GM077175) to L.T. Funding to pay the Open Access publication charges for this article was provided by the Wellcome Trust.

## Supplementary Material

Supplementary Data
