# Supplementary material for: A human mitochondrial poly(A) polymerase mutation reveals the complexities of post-transcriptional mitochondrial gene expression
Source: Hum Mol Genet. 2014 Jul 9;23(23):6345–55. doi: 10.1093/hmg/ddu352 (PMC4222368; doi:10.1093/hmg/ddu352)
Supplement: Supplementary Data [file supp_23_23_6345__index.html]

A human mitochondrial poly(A) polymerase mutation reveals the complexities of post-transcriptional mitochondrial gene expression — A human mitochondrial poly(A) polymerase mutation reveals the complexities of post-transcriptional mitochondrial gene expression — Supplementary Data 

# A human mitochondrial poly(A) polymerase mutation reveals the complexities of post-transcriptional mitochondrial gene expression

## Supplementary Data

Supplementary Data

**Files in this Data Supplement:**

- Supplementary Data - Pdf file
