# Supplementary material for: A human mitochondrial poly(A) polymerase mutation reveals the complexities of post-transcriptional mitochondrial gene expression
Source: Hum Mol Genet. 2014 Jul 9;23(23):6345–55. doi: 10.1093/hmg/ddu352 (PMC4222368; doi:10.1093/hmg/ddu352)
Supplement: Supplementary Data [file supp_ddu352_ddu352supp.pdf]

Supplemental Information

A human mitochondrial poly(A) polymerase mutation reveals the complexities of post-transcriptional mitochondrial gene expression

William C. Wilson, Hue-Tran Hornig-Do, Francesco Bruni, Jeong Ho Chang, Alexis A. Jourdain, Jean-Claude Martinou, Maria Falkenberg, Henrik Spåhr, Nils-Göran Larsson, Richard J. Lewis, Lorraine Hewitt, Arnaud Baslé, Harold E. Cross, Liang Tong, Robert R. Lebel, Andrew H. Crosby, Zofia M.A. Chrzanowska-Lightowlers* and Robert N. Lightowlers*

* Corresponding authors

Contact Information

Robert.Lightowlers@ncl.ac.uk +44 191-208-8028

zofia.chrzanowska-lightowlers@ncl.ac.uk +44 191-208-8028

Index

Supplemental Methods: page 2

Supplemental Figure Legends: page 3

Supplemental Figures:

Figure S1 page 5

Figure S2 page 6

Figure S3 page 7

Figure S4 page 8

Supplemental Methods:

Cell Proliferation Assays

Fibroblasts were seeded 25,000 cells/well in triplicate for each time point in 24-well plates. For cell growth curves in standard media containing glucose, cells were harvested and counted on specific days of the time course. For growth measurement in conditions forcing oxidative respiration, after seeding and incubating overnight, the standard culture media was replaced with DMEM medium lacking glucose (Sigma) supplemented with 10% (v:v) dialyzed FCS, 0.9mg/ml galactose, 0.11mg/ml sodium pyruvate, 1x non-essential amino acids, 50U/ml Penicillin, 50µg/ml Streptomycin, and 50µg/ml uridine. Cells were then harvested and counted at appropriate time points.

Analysis of mtPAP Dimerization by Size-exclusion Chromatography

Calibration standards (GE Healthcare) were prepared according to the manufacturer's instructions and injected onto a Superdex 200 HR 10/30 analytical size exclusion chromatography column (GE Healthcare), connected to an AKTA platform (GE Healthcare). The column was pre-equilibrated with buffer a consisting of 50mM Tris pH 8.5, 0.3M NaCl, a flow rate of 0.1ml/minute, and a column pressure of 0.5 MPa. The elution of proteins was monitored by UV absorbance. After elution of the calibration standards, wild-type and N478D recombinant mtPAP proteins were diluted to 0.45mg/ml in 200ul of the equilibration buffer, and 100ul of each injected onto the column separately. The elution fractions of the mtPAP proteins were compared to the elution fractions of the calibration standards to ascertain dimerization status.

Supplemental Figure Legends:

Figure S1. The 1432A>G mutation impairs growth on respiratory substrates. Patient and control fibroblasts (25,000 cells) were seeded in triplicate in glucose-based (left panel) or galactose-based (right panel) growth media, and cultured for 8 and 10 days, respectively. Cells were counted at the indicated time points. The mean value is plotted, n =3, error bars indicate ±SD.

Figure S2. Lentiviral expression of wildtype mtPAP restores polyadenylation and expression of mitochondrial gene products.

(A) Cell lysates (40μg) isolated from patient (P1, P2 – homozygous patients, lanes 2 and 3) and control (C, lane 4; Het – heterozygote, lane 1) fibroblasts expressing *PAPD1* transgene (+LVPAPD1) were separated via 12% SDS-PAGE. Representative western blot analysis depicts the levels of OXPHOS proteins using the antibodies indicated. The levels of mtPAP were determined using an antibody to the endogenous protein, which also detected the differently migrating FLAG tagged version.

(B) Steady-state levels of mitochondrial mRNAs were analysed by northern blotting (4μg RNA/ lane) following lentiviral transduction (+LVPAPD1) of control cell lines (C) and those carrying the 1432A>G mutation. Radiolabelled probes were used to the transcripts indicated. Mitochondrial rRNAs and cytosolic 18S rRNA were used as non-polyadenylated or loading controls, respectively.

(C) MPAT analysis of *MTND3* was performed on the cell lines described in (A), pre (-LVPAPD1; lanes 1-4) and post (+LVPAPD1; lanes 5-8) lentiviral transduction with wild type mitochondrial poly(A) polymerase. Poly(A) and oligo(A) populations are indicated following separation by denaturing PAGE (left panel). Densitometric profiles (ImageQuant software) of each lane are presented on the right indicating the relative amounts of poly- versus oligo-adenylated transcript.

*Figure S3. The p.*N478D *mutation does not affect mtPAP dimerization.*
Wild type and mutant recombinant mtPAP (4.5μg) were analysed by size exclusion chromatography. As size markers the elution fractions for ferritin (440kDa), aldolase (158kDa) and conalbumin (45kDa) are indicated.

Figure S4. Enhancement of poly(A) activity exerted by LRPPRC/SLIRP is sequence independent.
Wild type mtPAP (0.55μM) was incubated with (lanes 5 and 6) or without (lanes 2 and 3) LRPPRC/SLIRP complex (0.48μM). The RNA substrate (0.25μM) corresponded to a 40nt sequence internal of the *MTND3* transcript. Reactions proceeded for 60min or 120min at 37^{0}C before being quenched with 90% formamide/1x TBE, separated through a 15% polyacrylamide/8.3M urea gel and stained with SYBR gold. Stained products were visualized by scanning with a Typhoon FLA 9500 instrument.

Figure S1

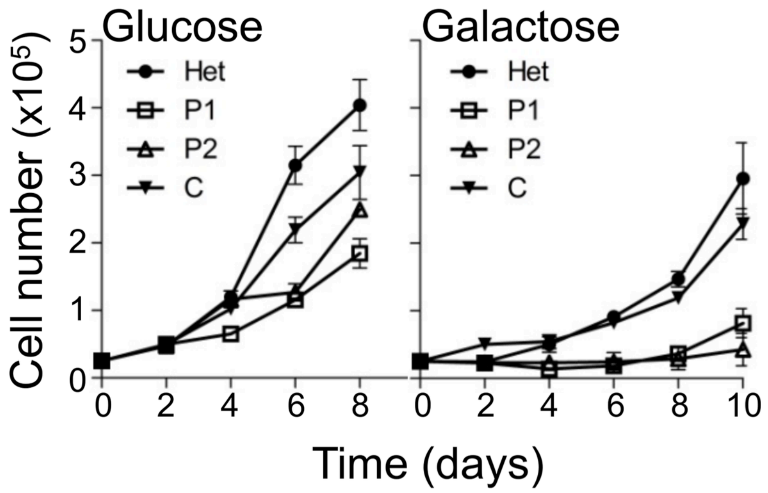
Glucose
Galactose
Het
P1
P2
C
Cell number (x10^5)
0
1
2
3
4
5
0
2
4
6
8
10
Time (days)

Figure S2

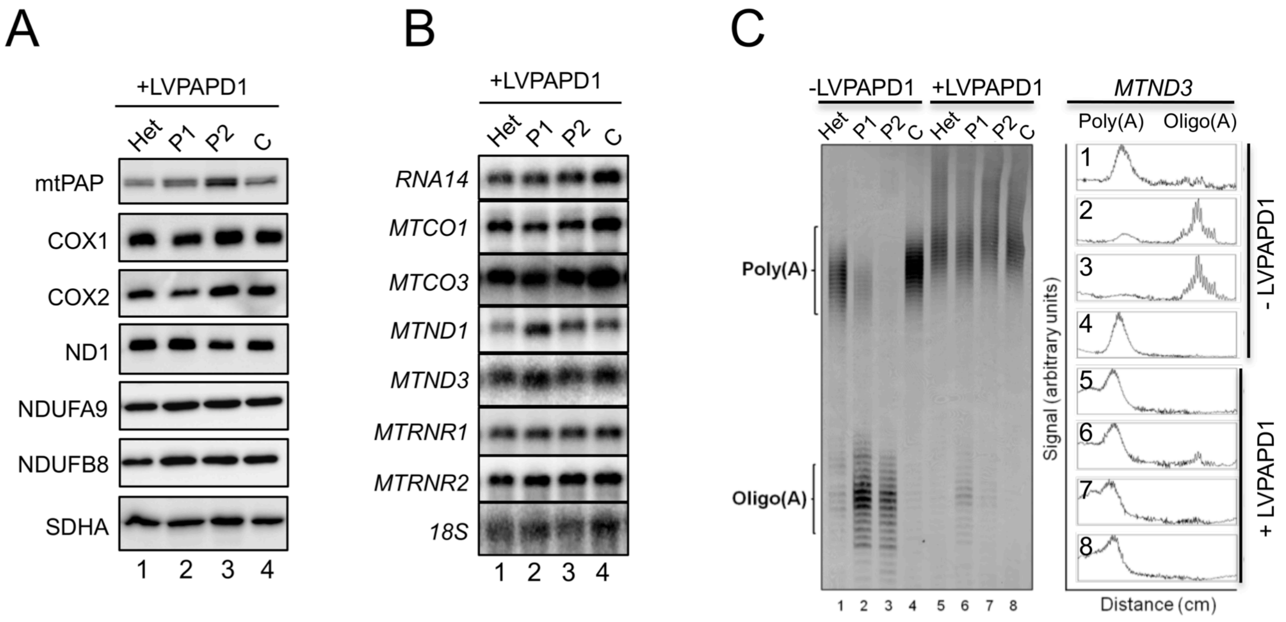
A
+LVPAPD1
Het
P1
P2
C
mtPAP
COX1
COX2
ND1
NDUFA9
NDUFB8
SDHA
1 2 3 4
B
+LVPAPD1
Het
P1
P2
C
RNA14
MTCO1
MTCO3
MTND1
MTND3
MTRNR1
MTRNR2
18S
1 2 3 4
C
-LVPAPD1
+LVPAPD1
Het
P1
P2
C
Het
P1
P2
C
Poly(A)
Oligo(A)
1 2 3 4 5 6 7 8
MTND3
Poly(A)
Oligo(A)
1
2
3
4
5
6
7
8
Signal (arbitrary units)
Distance (cm)
- LVPAPD1
+ LVPAPD1

Figure S3

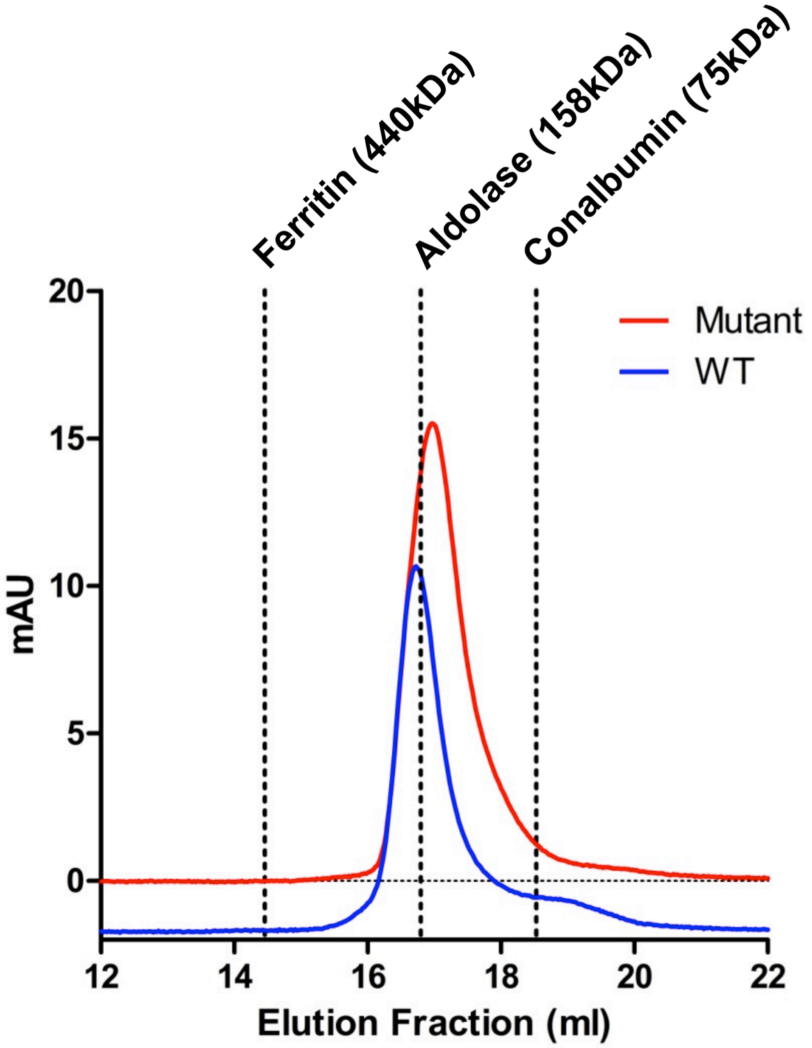

Size exclusion chromatography

Figure S4

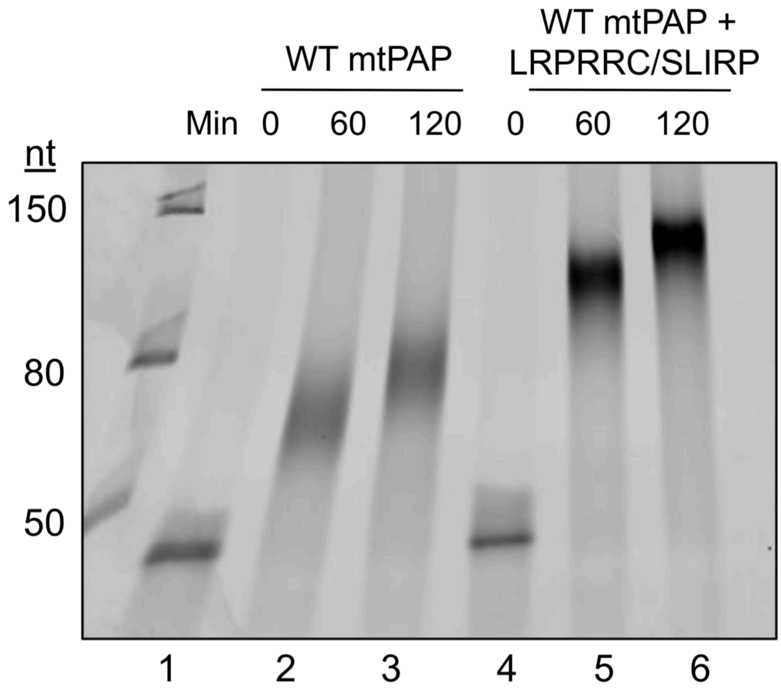

WT mtPAP
WT mtPAP +
LRPRRC/SLIRP
Min 0 60 120 0 60 120
nt
150
80
50
1 2 3 4 5 6
